# Supplementary material for: KSHV episomes reveal dynamic chromatin loop formation with domain-specific gene regulation
Source: Nat Commun. 2018 Jan 4;9:49. doi: 10.1038/s41467-017-02089-9 (PMC5754359; doi:10.1038/s41467-017-02089-9)
Supplement: Supplementary file 1 — Supplementary Information [file 41467_2017_2089_MOESM1_ESM.pdf]

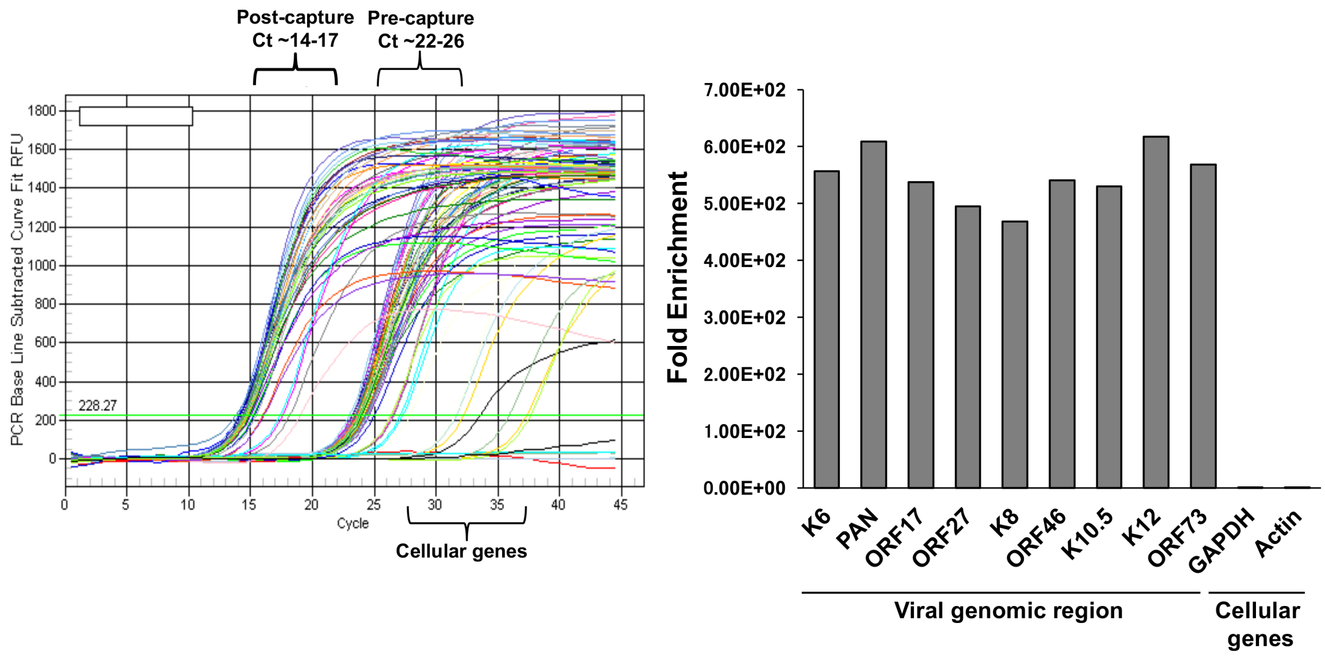

Supplementary Figure 1. KSHV 3C DNA enrichment. Following enrichment, equal amounts of pre- and post-enrichment DNA were analyzed by qPCR using primer pairs against several viral ORFs. Cellular GAPDH and ACTB served as specificity controls. The pre-capture cycle threshold for the viral genes clustered ~Ct of 22-26 while the post-capture samples clustered ~14-17. Reactions for cellular genes had pre-capture Ct of ~28 (GAPDH) or ~33 ACTB. All cellular genes had post-capture Ct >32. Fold enrichment was calculated using pre-capture cellular GAPDH Ct as the reference for both pre- and post-capture viral gene Ct values. These qPCR-based validation analyses demonstrated that the capture achieved approximately 600-fold enrichment of KSHV genomic sequences (*e.g.*, K6, PAN, and seven others) evenly across the genome, while cellular genomic sequences (*e.g.*, GAPDH, beta-actin) were not enriched.

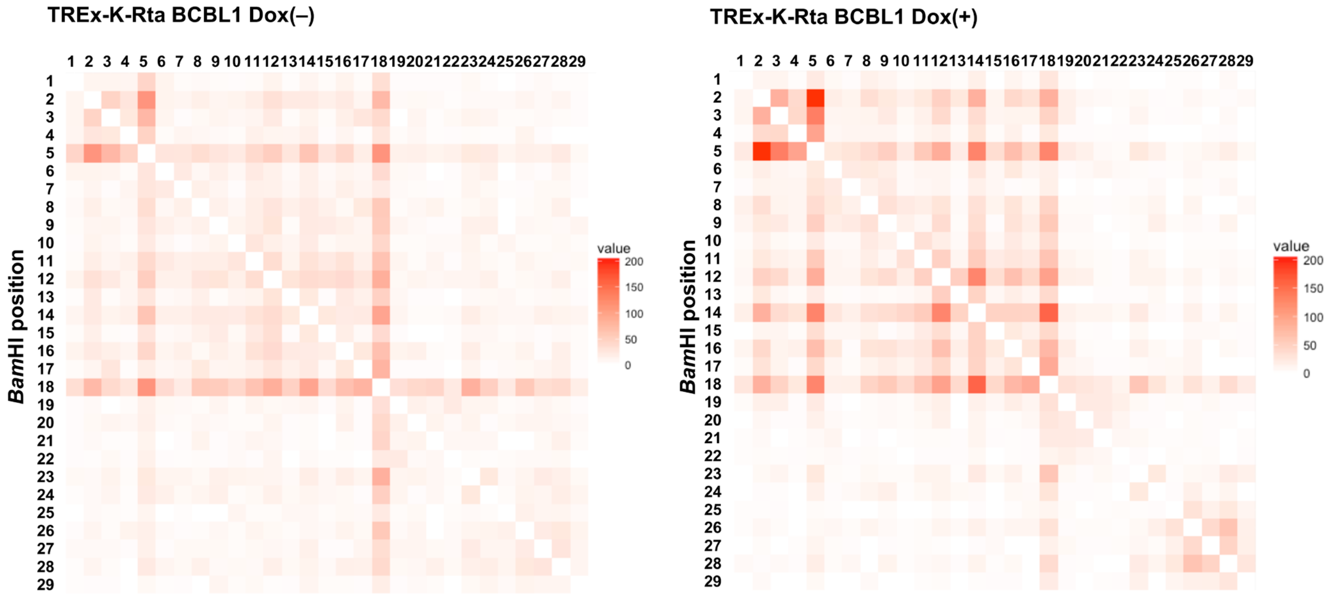

Supplementary Figure 2. Analyses of KSHV genomic loop formation with Capture Hi-C. TREx-K-Rta BCBL-1 cells were cultured without dox (-) or with dox (+) 1 µg/ml for 24 h and Capture Hi-C was carried out as described in the Methods. (a) Genomic loops heat map of KSHV genome before (left) and after (right) of K-Rta induction (24 h). Sequence reads corresponding to the sum of inward and outward at each genomic link were counted and visualized as heat maps with R software. The indicated positions of the 29 *Bam*HI sites are shown in the Circos maps in Figure 2a.

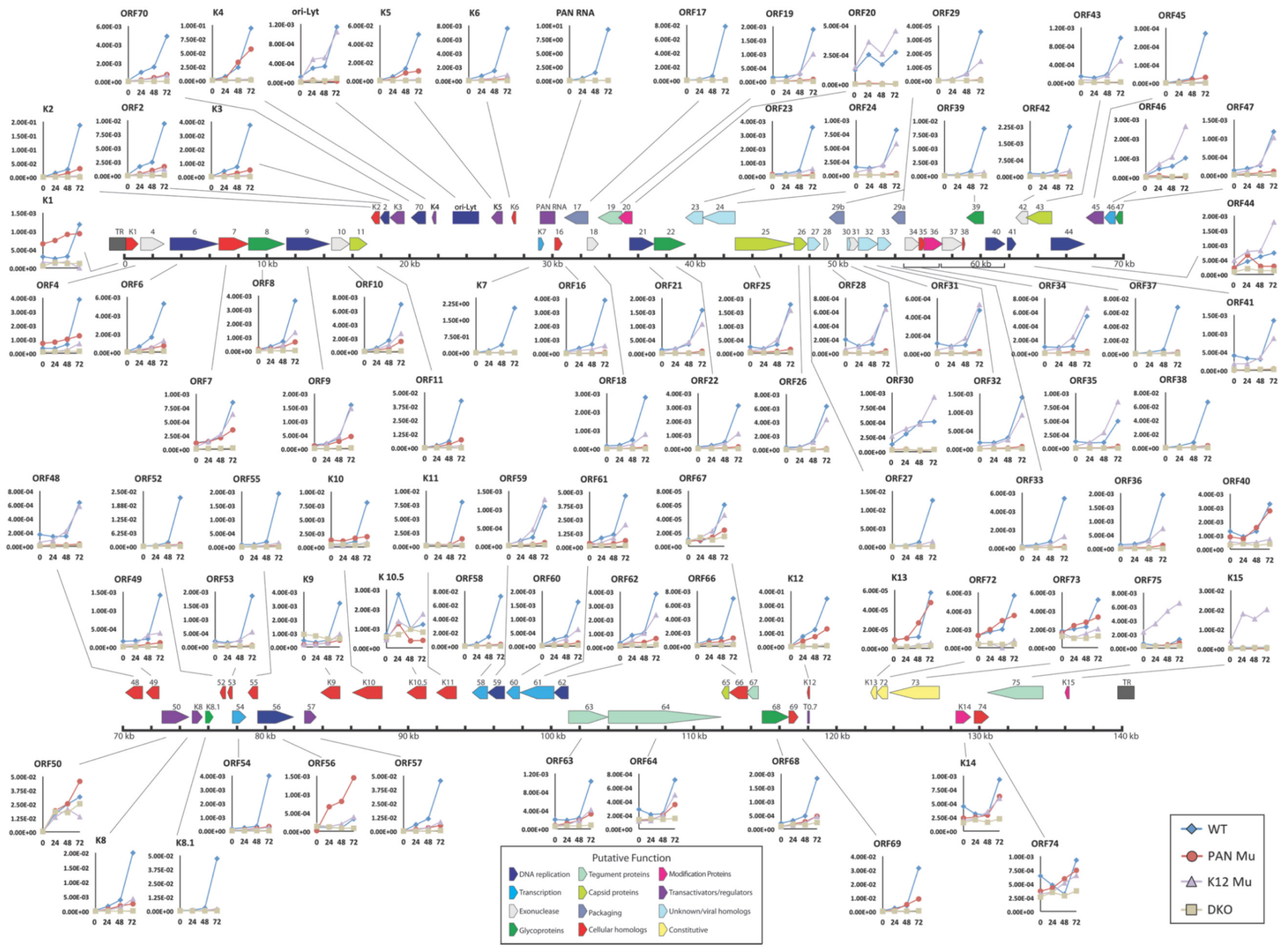

Supplementary Figure 3. Comprehensive picture of KSHV gene expression. Time course of KSHV gene expression in *i*SLK BAC16 cells lines; individual ORFs; WT (blue diamonds), PAN Mu (red circles), K12 Mu (grey triangles) or DKO (grey squares). Values are mean normalized expression (MNE) using ACTB expression as reference. A KSHV ORF map with putative function listed is shown in the diagram.

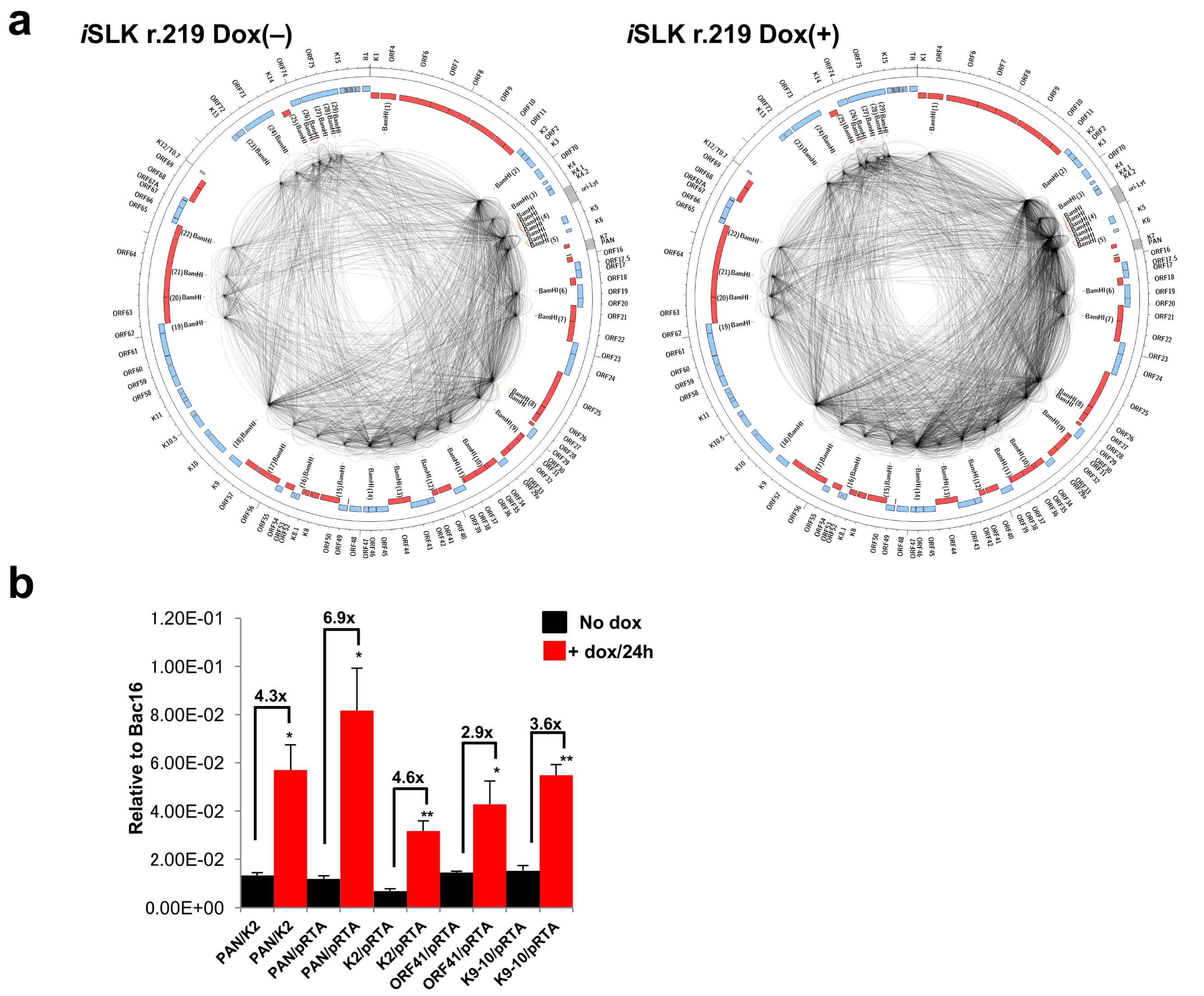

Supplementary Figure 4. Analysis and validation of KSHV genomic loop formation in *i*SLK/r.219 cells. (a) Capture Hi-C. *i*SLK/r.219 cells were cultured without Dox (-) or with Dox (+) 1 µg/ml for 24 h and Capture Hi-C was carried out as described in the Methods. Circos diagram depicting KSHV genomic links detected by Capture Hi-C before (left) and after (right) K-Rta induction. Values represent mean ± SD (n=3). **(b)** Validation of Capture Hi-C results. Enriched Capture-C DNA (*Bam*HI ligation products) was analyzed by qPCR for the contacts listed using DNA from *i*SLK/r.219 cells -/+ dox. Values represent signals obtained relative to a Bac16 *Bam*HI random ligation matrix. Fold increase (+dox/-dox) is listed. (* P<0.05; ** P<0.01, Student's t-Test).

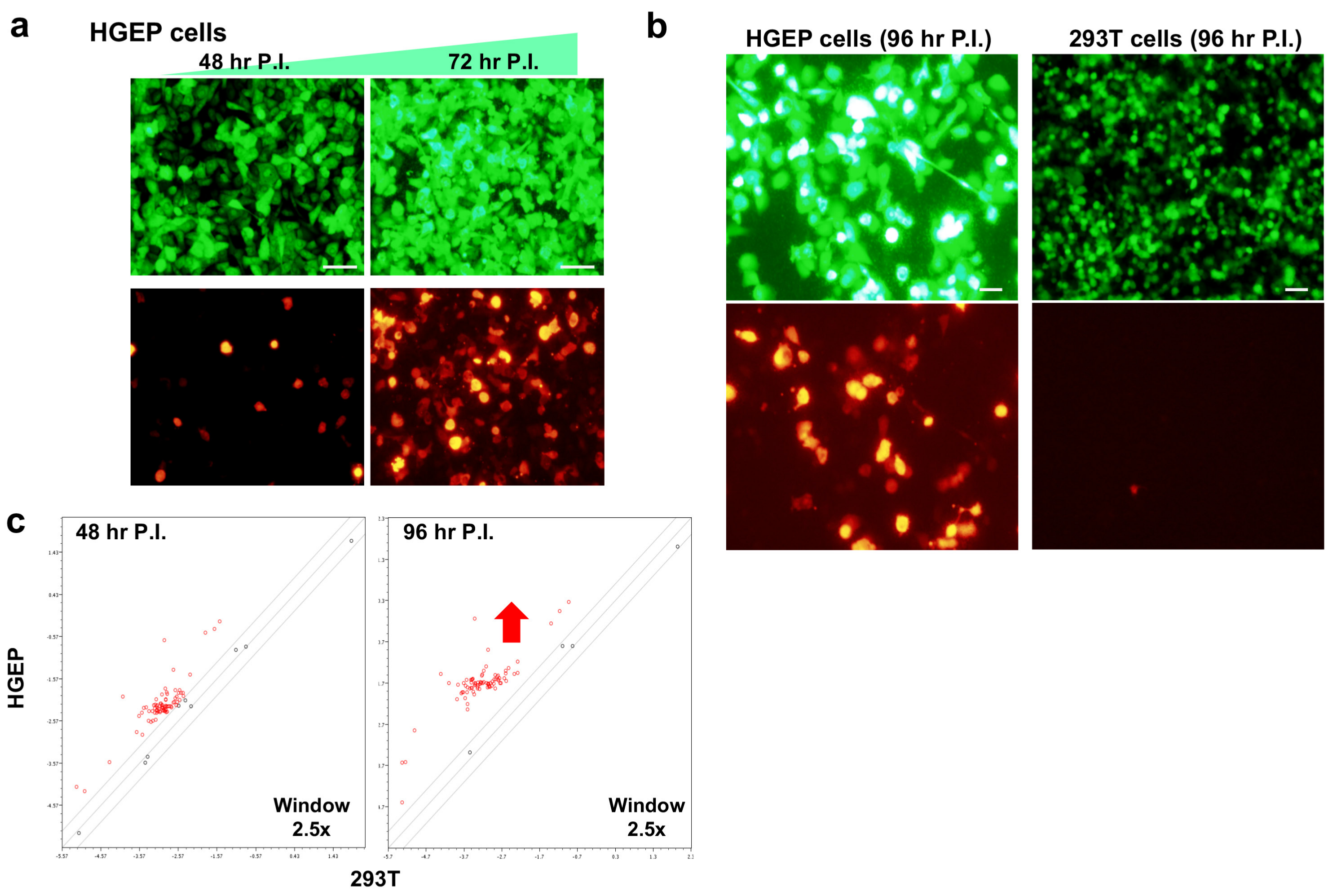

Supplementary Figure 5. ***De novo*** **infection of oral epithelial cells (a)** HGEP cells were infected (M.O.I. =10) for the indicated times with rKSHV.219 virus. Images were captured for eGFP (infected cells) and RFP (lytic cycle) expression with same exposure settings. Scale = 100 µm **(b)** HGEP and 293T cells were infected as in (A) and examined for eGFP/RFP expression at 96 h P.I. Scale = 20 µm (c) Scatter plots depicting KSHV gene expression at the indicated time points PI. Plots compare HGEP infected cells (y-axis) versus 293T infected cells (x-axis). 2.5x window; ACTB normalized.

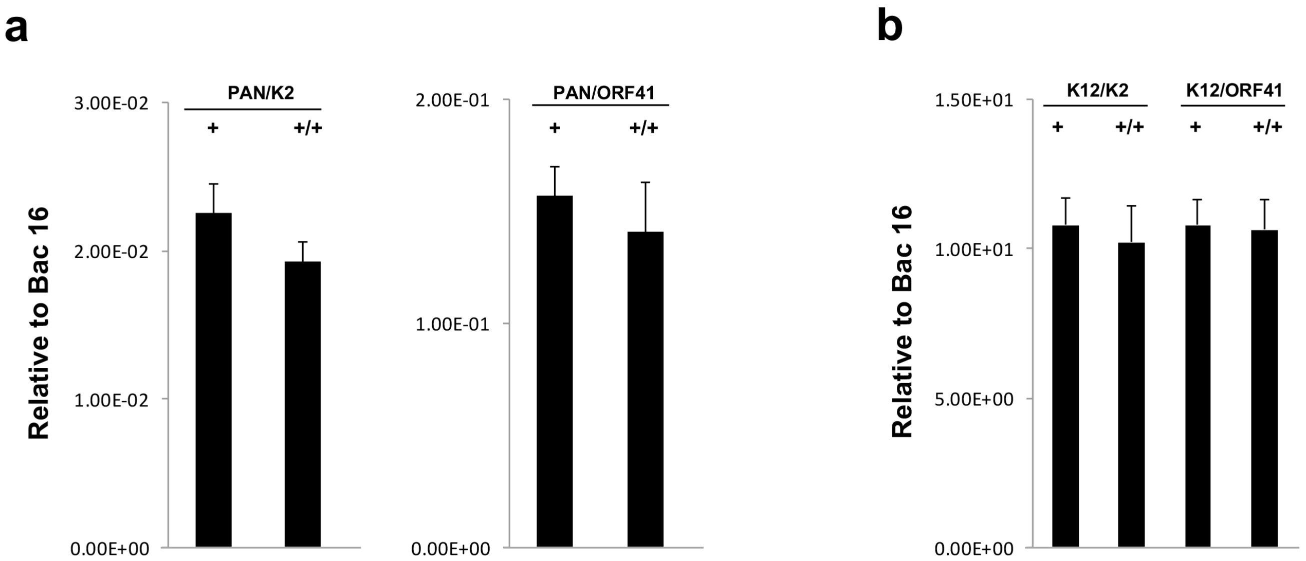

Supplementary Figure 6. Regulation of inducible loop formation by viral DNA replication. BCBL-1 TREx-K-Rta cells were treated with 1 µg/ml dox (+) or with 1µg/ml dox and 500 µM phosphonoacetic acid (PAA) (++) for 24 h. Dox and PAA were added to cell cultures simultaneously. At 24 hours cells were processed for 3C DNA as described in the Methods. qPCR results are shown relative to signal obtained from Bac16 *Bam*HI random ligation matrix. Genomic loops strongly induced during reactivation were selected for analysis. Values represent mean ± SD (n=3).

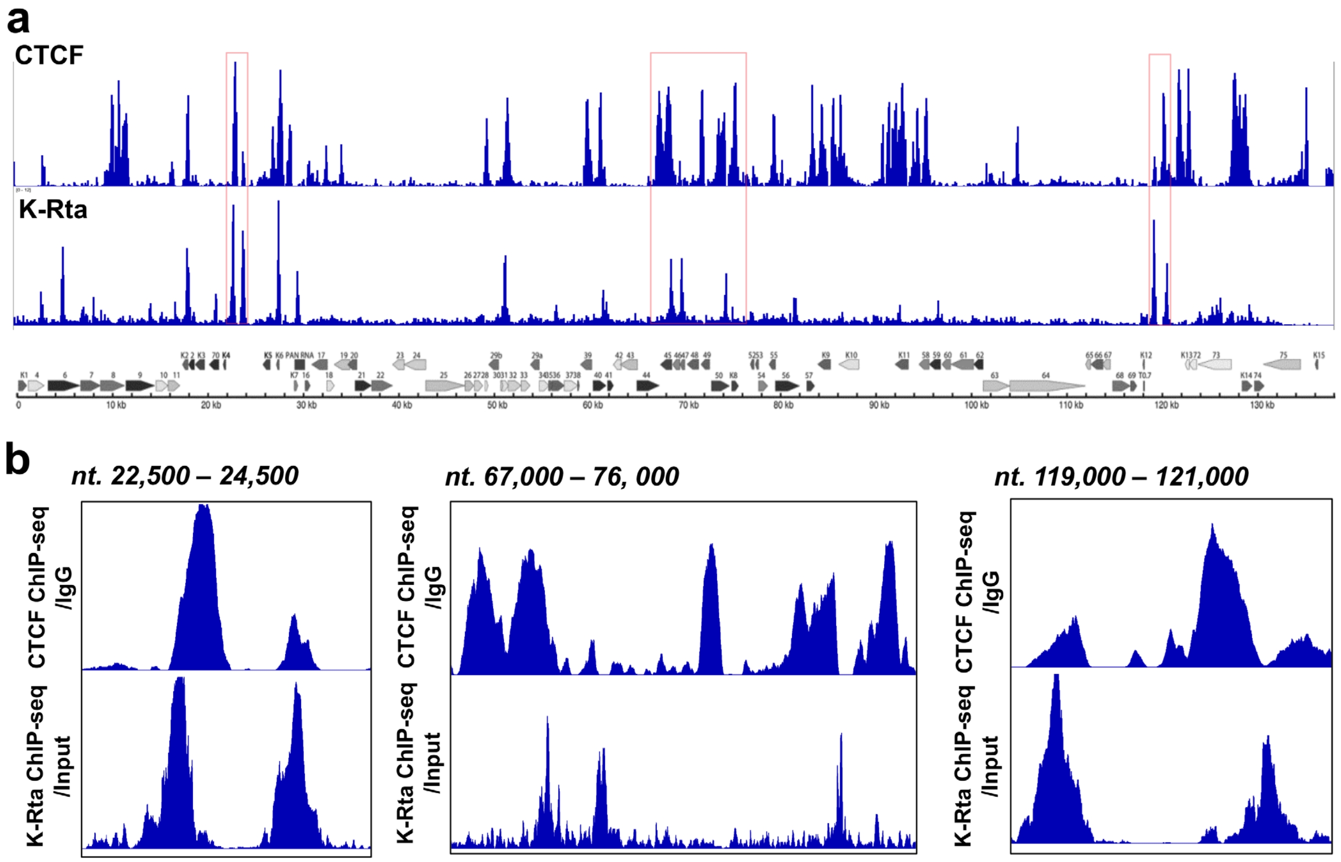

Supplementary Figure 7. KSHV genome wide K-Rta and CTCF recruitment sites. (a) Genome wide distribution of K-Rta and CTCF (GEO accession number: GSM941710) on the KSHV genome are shown. **(b)** Genome browser tracks showing zoom-in views of CTCF and K-Rta binding at the (left) Ori-Lyt-L, (center) Immediate-early, and (right) Ori-Lyt-R regions on the KSHV genome.

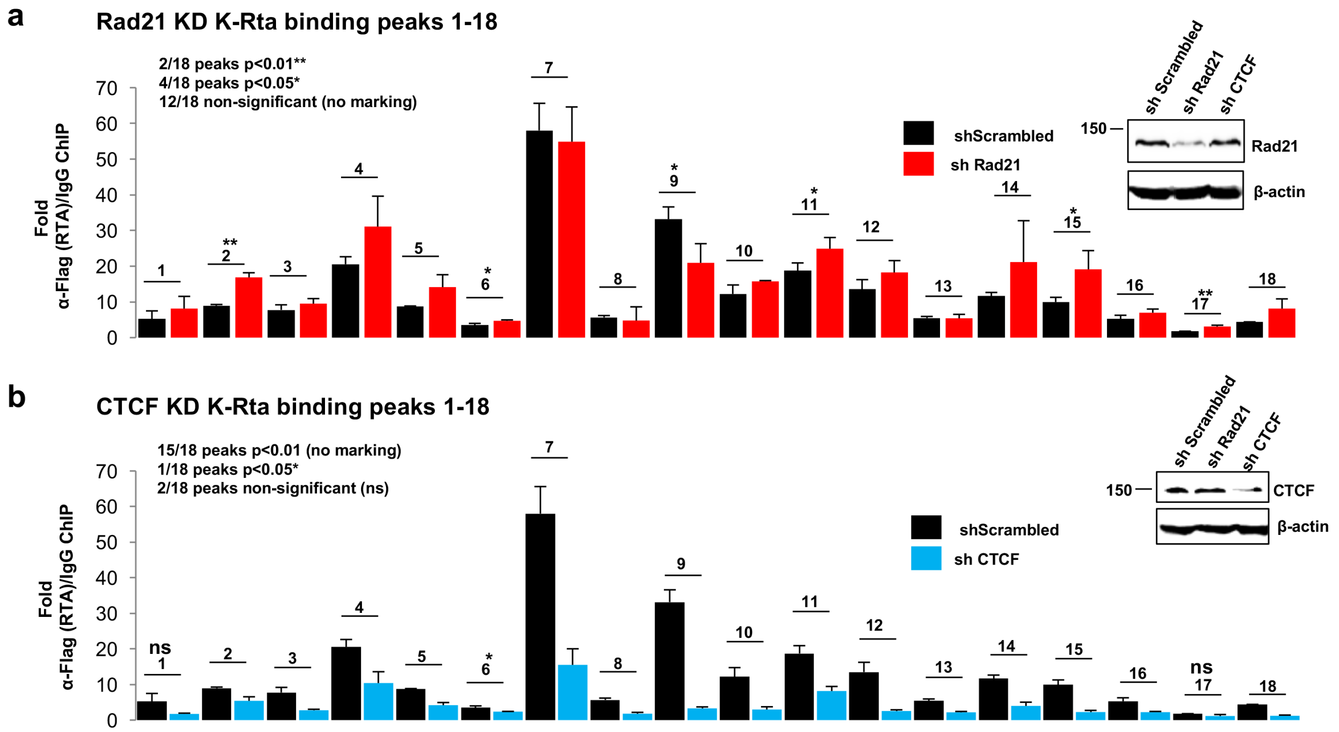

Supplementary Figure 8. Effect of Rad21 or CTCF Knockdown (KD) K-Rta binding to the KSHV genome. Lentiviral vectors were used to KD (a) Rad21 or (b) CTCF in BCBL-1 TREx-K-Rta cells for 72 h prior to dox-induced reactivation for 24 h. Sh Scrambled was used as control KD. KD constructs were obtained from Sigma (Mission shRNA). After 24 h of reactivation, cells were fixed and processed for ChIP as described in the Methods. Prior to fixation, a small number of cells were removed from each culture and processed for IB to evaluate KD efficiency. Blots were probed with anti-Rad 21 (Cell Signaling #12673) or CTCF (Cell Signaling #3417). ChIP DNA was prepared from anti-Flag (Sigma) or normal mouse IgG control antibody. For each ChIP, PCR primers were utilized to amplify sample DNA corresponding to each K-Rta enrichment peak listed in Table (1). Values are listed for each peak as Fold ChIP qPCR signal (anti-FLAG (Rta)/IgG control) (* $p<0.05$; ** $p<0.01$)

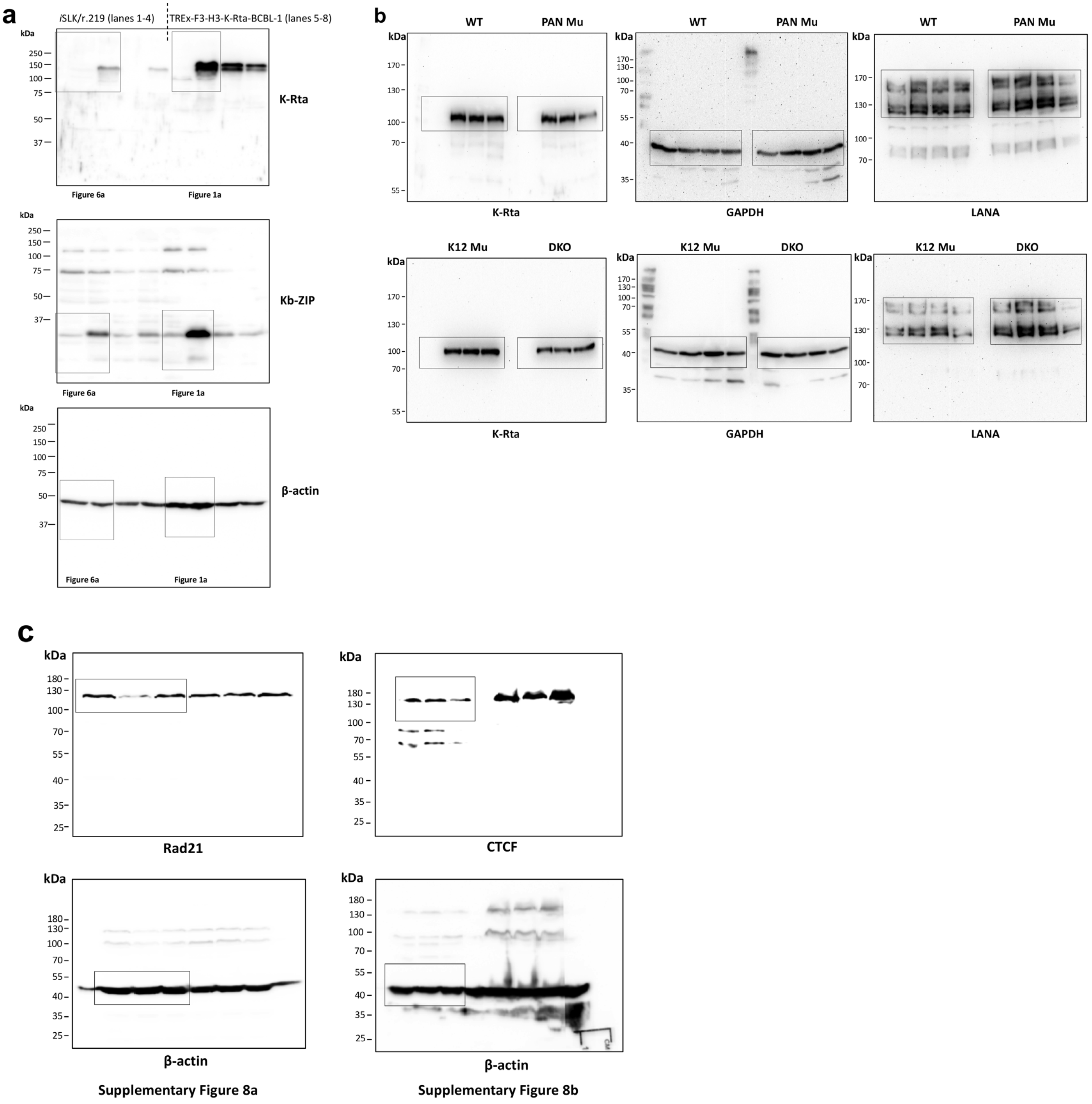

Supplementary Figure 9. Uncropped immunoblotting images. Uncropped images presented in **(a)** Fig. 1a and 6a, **(b)** Fig. 4b, and **(c)** supplemental Fig. 8a, b are shown.

Supplementary Table 1. K-Rta Recruitment sites

Peak Number	Genomic location (Reference to JSC1 BAC16)	Nearest ORFs or non-coding RNAs
1	2833-3177	ORF6
2	5000-5400	ORF7
3	8340-8551	ORF8
4	17940-18500	K2
5	21000-21350	ORF70-K4
6	Ori-Lyt-L	Ori-RNA
7	27560-27996	K5-K6
8	28500-30000	PAN RNA
9	51260-51767	ORF31-32
10	61560-62090	ORF41
11	68570-69190	ORF45
12	69620-70276	ORF47 (K-Rta promoter)
13	74360-74657	K-bZIP
14	81530-82000	ORF57
15	117900-118600	K12
16	Ori-Lyt-R	-
17	126000-126500	ORF73
18	134500-135500	ORF75

K-Rta recruitment sites were identified by ChIP-seq analysis of doxycycline-induced TREx-K-Rta BCBL-1 cells (Figure 1b), and are summarized. As described in *Methods*, the ChIP-Seq data was aligned to the reference KSHV genome sequence (GenBank: GQ994935.1) with Bowtie 2 and peaks identified by MACS2 are listed in the table.

Supplementary Table 2. 10 variants discovered by ultra-deep sequencing

				Bac16 WT		Bac16 PAN Mu		Bac16 K12 Mu		Bac16 DKO	
#CHROM	POS	REF	ALT	Number of high-quality bases	Number of high-quality non-reference bases	Number of high-quality bases	Number of high-quality non-reference bases	Number of high-quality bases	Number of high-quality non-reference bases	Number of high-quality bases	Number of high-quality non-reference bases
gi|261853473|gb|GQ994935.1|	24710	C	T	626	249	182	73	607	219	709	267
gi|261853473|gb|GQ994935.1|	28555	G	T	1517	1	307	307	1371	0	1155	1155
gi|261853473|gb|GQ994935.1|	28559	A	C	1542	0	282	282	1395	1	1053	1053
gi|261853473|gb|GQ994935.1|	28562	T	G	1544	0	269	269	1412	0	1004	1004
gi|261853473|gb|GQ994935.1|	28563	G	T	1532	0	269	269	1402	0	1006	1005
gi|261853473|gb|GQ994935.1|	28564	T	G	1531	1	272	272	1407	1	1027	1027
gi|261853473|gb|GQ994935.1|	119281	A	C	1688	1	467	0	1398	1392	1562	1561
gi|261853473|gb|GQ994935.1|	119286	T	G	1672	1	475	0	1403	1397	1552	1552
gi|261853473|gb|GQ994935.1|	119290	C	A	1671	0	471	0	1399	1393	1560	1559
gi|261853473|gb|GQ994935.1|	119316	A	G	1717	0	473	0	1467	1464	1680	0

The total number of reads at a given variant location are reported for each sample. Also included are the total number of reads matching to the alternate allele. Raw sequence reads were aligned back to the reference (Human herpesvirus 8 strain JSC-1 clone BAC16, GQ994935.1) using BWA-mem with default settings. SAMtools was then used to perform variant calling. Ultra-high coverage was obtained for most samples (~1200X) and allowed for comparative whole genome sequence analysis which revealed an extremely high degree of homogeneity between samples. A total of 10 variants were discovered.
